# An Oral Recombinant Vaccine in Dogs against *Echinococcus granulosus*, the Causative Agent of Human Hydatid Disease: A Pilot Study

**DOI:** 10.1371/journal.pntd.0000125

**Published:** 2008-01-16

**Authors:** Anne-Francoise Petavy, Carlos Hormaeche, Samia Lahmar, Hammou Ouhelli, Alejandro Chabalgoity, Thierry Marchal, Samira Azzouz, Fernanda Schreiber, Gabriela Alvite, Marie-Elisabeth Sarciron, Duncan Maskell, Adriana Esteves, Georges Bosquet

**Affiliations:** 1 Department of Parasitology and Medical Mycology, Lyon University, Lyon, France; 2 University of Cambridge, Cambridge, United Kingdom; 3 National Veterinary School, Sidi Thabet, Tunisia; 4 Institut Agronimique et Vétérinaire Hassan II, Rabat, Morocco; 5 Facultad de Medicina y Facultad de Sciencias, University Montevideo, Montevideo, Uruguay; 6 Veterinary School, Lyon, France; Fundação Oswaldo Cruz, Brazil

## Abstract

Dogs are the main source of human cystic echinococcosis. An oral vaccine would be an important contribution to control programs in endemic countries. We conducted two parallel experimental trials in Morocco and Tunisia of a new oral vaccine candidate against *Echinococcus granulosus* in 28 dogs. The vaccine was prepared using two recombinant proteins from adult worms, a tropomyosin (EgTrp) and a fibrillar protein similar to paramyosin (EgA31), cloned and expressed in a live attenuated strain of *Salmonella enterica* serovar *typhimurium*.

In each country, five dogs were vaccinated with the associated EgA31 and EgTrp; three dogs received only the vector *Salmonella*; and six dogs were used as different controls. The vaccinated dogs received two oral doses of the vaccine 21 d apart, and were challenged 20 d later with 75,000 living protoscoleces. The controls were challenged under the same conditions. All dogs were sacrificed 26–29 d postchallenge, before the appearance of eggs, for safety reasons.

We studied the histological responses to both the vaccine and control at the level of the duodenum, the natural localization of the cestode. Here we show a significant decrease of parasite burden in vaccinated dogs (70% to 80%) and a slower development rate in all remaining worms. The *Salmonella* vaccine EgA31-EgTrp demonstrated a high efficacy against *E. granulosus* promoting its potential role in reducing transmission to humans and animals.

## Author Summary

In many countries in the world, livestock and humans are affected with hydatid disease, which is caused by the development, in the viscera, of the larval stage of the cestode *Echinococcus granulosus*. They become infected by ingesting the eggs of this parasite, which are passed in the feces of the dog—the host of the adult worm. Domestic dogs are key in the transmission to livestock and humans.

This disease remains a major economic and public health problem in affected countries. Because dogs are quickly reinfected, control programs in these locations include monthly anthelmintic deworming. These control measures, however, are burdensome for the owner, so they often fail. In contrast, vaccination can take place in control programs at different stages of the parasite life cycle. For example, currently an effective recombinant vaccine for sheep has been developed that should work indirectly to reduce infection in dogs, which tend to eat sheep offal. However, we propose that a recombinant oral vaccine given to the small number of dogs keeping the herd would decrease the number of *Echinococcus granulosus* adult worms and, consequently, the number of infective eggs. This measure would help reduce the contamination risk factors for humans and livestock, and would be cost-effective for the owners of the dogs.

## Introduction

Cystic echinococcosis, also called hydatidosis, represents a severe public health and livestock problem, particularly in developing countries [1]–[3]. The causative agent is the cestode *Echinococcus granulosus*. The adult stage may be found in the small intestine of canine carnivores. Growth of the larval stage throughout the internal organs, especially the liver and lungs, causes clinical signs in the intermediate hosts, such as sheep, cattle, and camels. Humans may also become host to this metacestode. Usually, however, intermediate hosts become infected by grazing on vegetation contaminated by eggs shed by adult worms via canine feces [4].

In various endemic areas, prevention and control programs have been established [5]. These programs usually involve the repeat treatment of dogs with praziquantel alongside the establishment of health education programs. However, such programs represent a significant financial burden for developing countries.

A vaccine against hydatidosis (the disease caused by the larval stage of the parasite), or echinococcosis (the disease caused by the adult stage), is thus highly desirable in order to provide long-term prevention of the disease and to complement control programs. An effective vaccine against ovine hydatidosis, based on a recombinant protein from parasite oncospheres (first larval stage of the parasite), has been developed that targets the larval stage of the parasite [6]. If used in the field, this vaccine would need to be administered to all animals in a herd, which may be very costly to control programs. In contrast, a vaccine directed at protecting dogs against the adult worm would have to be given to only a few animals to protect the environment, because dogs are less numerous than other animals in the herd. Mathematical modeling has recently confirmed the possible utility of this strategy [7], and several authors have demonstrated its feasibility by showing that dogs can develop protective immunity against *E. granulosus* [8],[9].

Based on these reports, subcutaneously injected anti-echinococcosis vaccines have been prepared from soluble native protoscolex (scoleces situated in brood capsules) proteins as well as from recombinant proteins normally expressed by mature adult worms [10]. This vaccine aims at decreasing worm growth and egg development.

Here we describe a vaccine that uses a live attenuated *Salmonella* mutant strain as a vector to deliver two recombinant proteins expressed by the adult stage of *E. granulosus*: tropomyosin (EgTrp) and (EgA31), which share some sequence elements with paramyosins. The vaccine is intended to protect domestic and stray dogs as well as wild canids from infection with *E. granulosus*. We studied the histological and immunological responses to both the vaccine and to a challenge at the level of the upper duodenum in dogs.

## Materials and Methods

### Plasmids and bacterial strains

EgA31 and EgTrp are recombinant proteins. As described by Saboulard et al. [11], a PstI/DraI cDNA fragment of the EgA31 cDNA (GenBank [http://www.ncbi.nlm.nih.gov/] accession no. AF067807) was cloned into the PQE80L expression vector (Qiagen). Plasmid pQE11-EgTrp encoding the C terminus of *E. granulosus* antigen EgTrp and plasmid pTECH2 1994 have been described elsewhere [12],[13].

*S. enterica* serovar *typhimurium* (*S. typhimurium*) aroC strain LVR01 suitable for oral immunization of dogs is described elsewhere [14].

### Cloning and expression of EgA31 and EgTrp as TetC fusions in the *Salmonella* vaccine strain

An immunogenic fragment encoding aa 168–246 [11] from EgA31 was amplified by PCR from pQE80[egA31] using the primers EgA3 (forward primer: 5′-CATGGATCCGCTGAAAAACAAGCCATGGAT-3′), and EgA4 (reverse primer: 5′-ATGAAGCTTAATTTCAGCTTTCTGCTC-3′). Forward and reverse primers contained BamHI and SpeI restriction sites (underlined) to facilitate directional cloning into pTECH as previously described [14].

The EgTrp (GenBank accession no. AAB65799) was amplified by PCR from pQE11-EgTrp using primers TrpF (forward primer: 5′-CGCGGATCCGAAACATCTACTAAGCTTGACG-3′), and TrpR (reverse primer: 5′-CCCAAGCTTTCAGAAGGAAGTGAGCTCCGCG-3′). Forward and reverse primers contained BamHI and SpeI restriction sites (underlined) to facilitate directional cloning into pTECH.

Each of the PCR products was ligated into the pTECH plasmid, which had been previously digested with the same enzymes, and the ligation product was transformed into *E. coli* strain TG2. Transformant colonies were evaluated by DNA restriction analysis of the plasmid. Expression of the TetC fusions was tested by Western blotting on lysates of bacteria harboring the construct, using anti-TetC serum and either anti-EgA31 or anti-EgTrp sera as probes, as previously described [15]. The constructs were then transferred to Salmonella LVR01 and tested again for expression of the fusion protein.

### Experimental animals

All work with dogs was conducted following international guidelines on the use of animals for experimentation (recommendation of the European Commission No L 358, ISSN 0378-6978). Dogs of common breeds, between 1 and 6 mo of age, were purchased locally in Tunisia and Morocco and kept in approved facilities for 2 mo before use.

A total of 28 dogs were used in this study, 14 in each country. Dogs were divided into four groups, with the number, sex, and age detailed in Table 1.

**Table 1:** Age, Sex, and Status of the Group of Dogs Used in the Experiments in Morocco and Tunisia

Group	Morocco			Tunisia		
	Dog Identification	Age-Sex	Status	Dog Identification	Age-Sex	Status
Group 1: Received EgA31-EgTrp expressed in *Salmonella*	11	5-F	Vaccinated	01	5-F	Vaccinated
	10	6-F	Vaccinated	04	5-M	Vaccinated
	07	3-M	Vaccinated	05	5- M	Vaccinated
	06	3-F	Vaccinated	06	6-F	Vaccinated
	04	3-M	Vaccinated	10	5-F	Vaccinated
Group 2: Received the vector not expressing the antigen	05	3-M	*Salmonella*	11	5-F	*Salmonella*
	16	3-F	*Salmonella*	12	5-F	*Salmonella*
	01	5-M	*Salmonella*	14	4-F	*Salmonella*
Group 3: Controls	08	3-F	Control PBS	03	6-F	Control PBS
	09	3-M	Control PBS	08	7-M	Control PBS
	19	3-F	Control PBS	02	4-M	Infected control
	13	3-F	Infected control	15	4-M	Infected control
	18	3-M	Infected control	37	10-M	Infected control
	16	5-M	Negative control	36	5-M	Negative control

Group 1: Ten animals. All were vaccinated with EgA31 and EgTrp, before being challenged with protoscoleces.

Group 2: Six animals. All received the vector *Salmonella* not expressing any *E. granulosus* antigen, before being challenged with protoscoleces.

Group 3: 12 animals. All were controls: Five dogs received a mock vaccination with 0.1 mM PBS before being infected with protoscoleces; five dogs were only infected with protoscoleces; and two dogs were the noninfected (negative) controls.

### Vaccination protocols and challenge

For oral immunization, dogs were starved 12 h before being allowed to ingest 5×10^10^ recombinant bacteria in 2 ml of PBS, or PBS alone as previously described [15]. Animals received two doses 21 d apart. Bacterial cultures were prepared just before each vaccination dose. Weekly blood samples were taken after immunization ,and sera were stored at −20°C until testing.

Twenty days after the last dose of *Salmonella,* all animals were orally challenged with 7.5×10^4^ live protoscoleces obtained from liver cysts recovered from sheep. The viability of protoscoleces was verified before challenge. Dogs were euthanized by intravenous injection of pentobarbital 26–29 d post-challenge.

### Tissue collection

Immediately following euthanasia, full-thickness sections of the experimental and control dogs' proximal duodenum (always within 10–15 cm from the pylorus) were collected for immunostaining and histological examination.

Worms were recovered by scraping the intestinal mucosa followed by several washings with 0.9 N NaCl solution and a series of sedimentation steps.

### Preparation for immunostaining and histological examination

Tissues were fixed in 10% neutral-buffered formalin, embedded in paraffin wax, sectioned at 6 µm, and either stained with haematoxylin for routine histological evaluation or transferred onto poly-l-lysine–pretreated slides for immunohistochemical studies.

To identify T cells and plasma cells in sections, we used a panel of primary antibodies to: CD3, lambda (λ), kappa (κ), IgA, IgM, as well as a standard avidin-biotin ABC immunoperoxidase (Autoprobe II Biomeda). Briefly, fixed sections were passed through graded alcohol to PBS (0.01 M [pH 7.2]), then lightly digested in stabilized enzyme mixture (Auto/Zyme Reagent Set; Biomeda) for 10 min at 37°C to break the disulphide bridges and enhance antigen retrieval. After one wash in PBS, sections were heated in 10 mM citrate buffer (pH 6.0) for 40 min at 90°C in a double boiler.

Endogenous peroxidase activity was blocked by incubation with hydrogen peroxide (3% v/v) in PBS for 10 min, and slides were then incubated for 10 min with a blocking solution (tissue conditioner, Biomeda) to reduce nonspecific background activity. Sections were incubated with primary antibody for 1 h and sequentially incubated with biotinylated secondary antibody (Autoprobe II, Biomeda) for 30 min. Prior to use, the secondary antibody was incubated for 30 min with 10% (v/v) dog serum. Slides were then incubated with streptavidin-biotin horseradish peroxidase complex (Autoprobe II, Biomeda) for 30 min. All incubations were performed at room temperature.

We used PBS to wash sections three times between each incubation step, to perform all dilutions, and to replace primary antibodies for control purposes.

Binding of the streptavidin-biotin conjugate was visualized by addition of 3,3′-diaminobenzidine terahydrochloride and hydrogen peroxide (Autoprobe II, Biomeda); sections were counterstained with haematoxylin.

### Examination of sections and labeled cell quantification

To ensure homogeneity of the analyses, all sections were coded and analyzed in a blinded fashion by the same investigator. Samples were examined with an Olympus BX 50 microscope.

For each section, we counted positively stained cells in the lamina propria of villi. Ten areas of the duodenal mucosa including one or more villi (except those just above Peyer's patches), were chosen at 20× objective, digitized with a Nikon Coolpix 4500 digital camera, and then transferred to a computer by means of Nikon software (Eclipse net). Lamina propria from each villus, from base to tip, was delineated on the computer screen (excluding epithelium and large vessels), and positively stained cells within each region were manually counted.

### Electron microscopy methods

Fragments (less than 1 mm thick) of the different intestinal segments were fixed in 2% glutaraldehyde–sodium cacodylate/HCl 0.1 M (pH 7.4) for 2 h at 4°C. After washing in socium cacodylate/HCl 0.2 M (pH 7.4), the samples were post-fixed in 1% OsO_4_–sodium cacodylate/HCl 0.15 M (pH 7.4) for 1 h at 4°C, and dehydrated in graded (from 30% to 100%) ethanol.

For transmission electron microscopy, samples were impregnated in Epon and flat-embedded in order to define the orientation of the tissue sample (epithelio-connective interface) for sectioning. After polymerization at 60°C for 3 d, thin sections were obtained on an RMC MTX ultramicrotome: semi-thin sections (1 µm thick) deposited on glass slides were stained according to Richardson et al. [16] with methylene blue and Azur II; and ultra-thin sections (60–80 nm) adhered to copper grids were contrasted according to Reynolds [17] with uranyl acetate and lead citrate. Sections were observed using a Jeol 1200 CX transmission electron microscope equipped with numerical camera SIS Megaview II. Iconography was treated with AnalySIS software.

### ELISA determination of antibody responses

96-well micro plates (Nunc-Maxisorb) were coated with 1 µg/ml of each recombinant protein in 30 mM carbonate-bicarbonate buffer (pH 9.6). After overnight incubation at 4°C, the plates were washed with 0.05% Tween 20 in PBS (PBS-Tween), and blocked with PBS-Tween containing 1% bovine serum albumin for 2 h at 37°C. Pools of dog serum made up in PBS-Tween were added to triplicate wells and the plates incubated at 37°C for 2 h. After three washes with PBS-Tween, 100 µl/well of each of the diluted specific antibodies (1∶100), the monoclonal goat anti-dog IgG (Sigma-Aldrich), IgA, and IgE (Interchim) were added. After incubation for 2 h at 37°C, the plates were washed, and 100 µl/well of peroxidase-labeled rabbit anti-goat IgG (Sigma-Aldrich) diluted 1∶5,000 in PBS-Tween was added. After incubation (1 h at 37°C), the plates were washed again, and the enzyme reaction developed with the substrate 3,3′,5,5′-tetramethylbenzene-dihydrochloride (Sigma-Aldrich). Optical densities were read at 450 nm with an ELISA plate reader (Microplate Reader, Bio-Rad Laboratories). Sera from the control dogs (nonvaccinated and noninfected) were pooled and used as a negative control to measure the background activity in all experiments.

### Statistical analysis

INSTAT software (GraphPad) was used for the statistical studies. One-way analysis of variance (ANOVA), and the Dunnett multiple comparisons post-test were employed. Statistical differences with *P*<0.05 were considered significant.

## Results

### Cestode counts

We counted the number of worms in all groups of animals. Total and average numbers are presented in Table 2. Results of the trial in Morocco showed a 79% reduction in the number of cestodes in vaccinated dogs compared with nonvaccinated, noninfected controls. Results did, however, vary among animals: one dog (number 4) from the vaccinated group had the largest worm load (unpublished data), suggesting that the vaccine did not exert any protective effect in this animal.

**Table 2:** Experiment Done in Morocco and Tunisia: Worms Counted in Each Group of Dogs

Group	Dogs (*n*)	Morocco		Tunisia	
		No. of Worms	Mean/Dog	No. of Worms	Mean/Dog
Vaccinated	5	2,825 (170–1,130)	565** #	3,904 (160–1,350)	650** #
*Salmonella* alone	3	4,495 (265–3,570)	1,498**	4,060 (508–2,100)	1,350**
Control	3	15,720 (390–8,640)	2,620	12,312 (160–6,500)	2,460

**: *P*<0.01, Dunnett multiple comparisons test. *P*-value as compared with dogs from control groups.

# *P*<0.05, Dunnett multiple comparisons test. *P*-value as compared with dogs that received only *Salmonella*.

Similar to those seen in Morocco, the results from the trial carried out in Tunisia showed a 74% reduction in the number of worms in vaccinated compared with control dogs (Table 2).

### Worm size variation

We measured the sizes of 50 randomly chosen worms per experimental group and recorded the percentage of developed (≥5 mm) versus underdeveloped (<5 mm) worms. In the vaccinated dogs, 40% of the worms were small, whereas in infected control dogs, small worms represented only 15% of the total population. We observed a similar distribution in the Tunisian experiments. It is worth noting here that a decrease in the number of parasites and a delay in their growth rate are considered to be two criteria for the effectiveness of a vaccine against the adult worm.

### Histological, immunostaining, and electron microscope results

Haematoxylin-stained sections were examined prior to immunostaining. Table 3 shows the number of cells/mm^2^ that stained positively with the antibodies studied in each group of dogs. For each antibody, we assessed a mean area of 0.58 mm^2^. The results from the vaccinated group exclude dog 4, which had the large parasite load.

**Table 3:** Number of Positive Cells per Square Millimeter after Immunostaining with Various Antibodies in the Villous Lamina Propria of the Duodenum

Antibodies	Vaccinated Dogs	Dogs Receiving only *Salmonella*	Control
CD3	591*	1,414	1,245
Lambda (λ)	1,912##	1,177	1,591
Kappa (κ)	339	297	324
IgA	1,460	1,048	1,395
IgM	189	193	217
λ+κ/CD3	3.81#	1.04	3.71
IgA+IgM/CD3	**2.79** ## ^*^	**0.88**	**1.45**

The average of Lambda positive cells was 1,594 per mm^2^ and of Kappa positive cells 322 per mm^2^; thus the λ/κ ratio is 5. The average of IgA positive cells was 1,335 per mm^2^ and of IgM positive cells 202 per mm^2^, thus the IgA/IgM ratio is 6.6.

*: *P*<0.05, Dunnett multiple comparisons test.

## *P*<0.01, Dunnett multiple comparisons test.

# *P*<0.05, Dunnett multiple comparisons test.

# *P*-value as compared with dogs who received only *Salmonella*.

**: *P*-value as compared with dogs from control groups.

**Figure 1:**
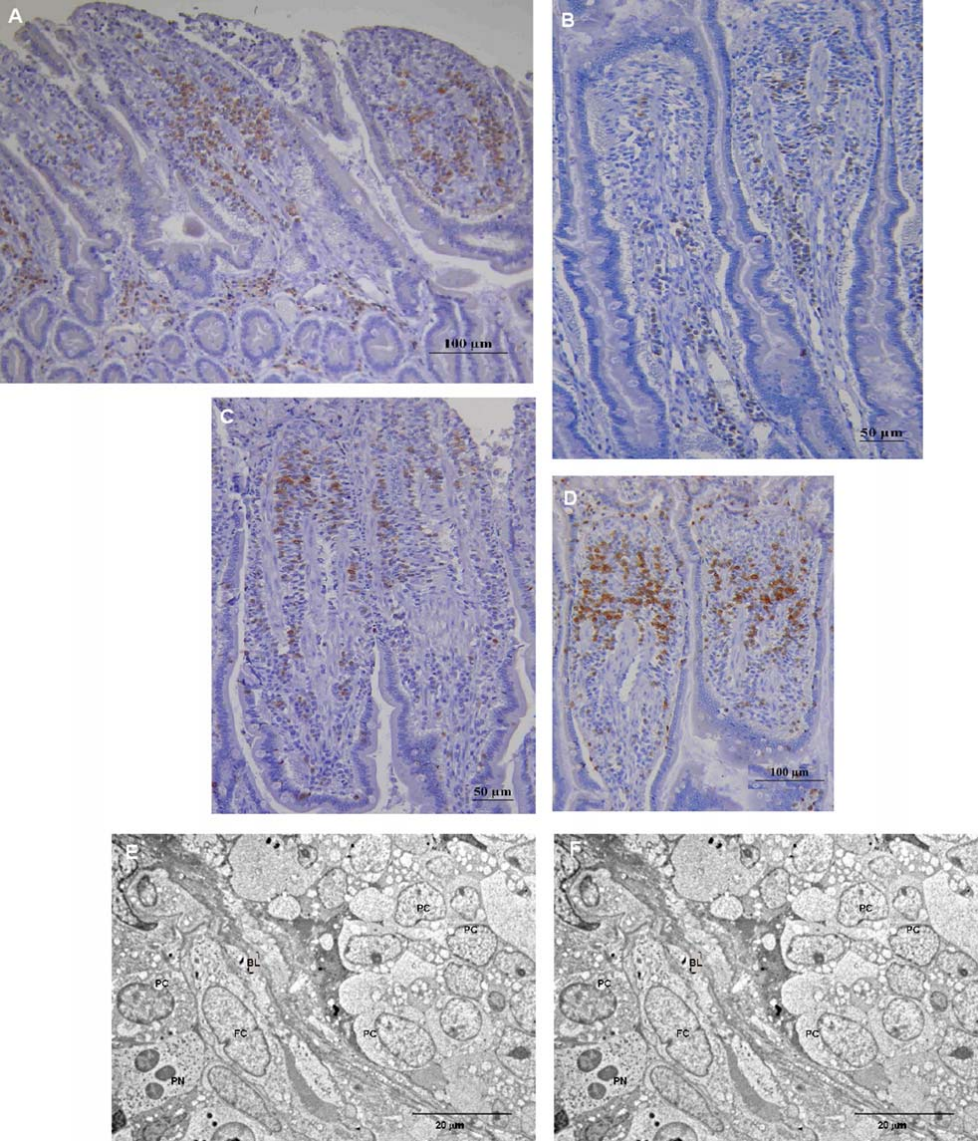
Immunocompetent Cell Responses at the Level of the Duodenum of Vaccinated and Control Dogs. (A) Duodenum of a vaccinated dog: The interstitial infiltrate of immunocompetent cells are labelled with a polyclonal rabbit antiserum specific for the Fc fragment from IgA of dog. IgA. Binding is visualized by the peroxidase reaction (brown cells) and hematoxylin counter stained. (B) Duodenum of an infected control dog: The interstitial infiltrate of immunocompetent cells are labelled with a polyclonal rabbit antiserum specific for the Fc fragment from IgA of dog. IgA. Binding is visualized by the peroxidase reaction (brown cells) and hematoxylin counter stained. (C) Duodenum of a vaccinated dog: T cells in the interstitium at the top of the villi are immunolabelled with rat antihuman CD3IgG1, clone number CD3-12. Binding is visualized by the peroxidase reaction (brown cells) and hematoxylin counterstained. (D) Duodenum of a control infected dog: T cells in the interstitium at the top of the villi are immunolabelled with rat antihuman CD3IgG1, clone number CD3-12. Binding is visualized by the peroxidase reaction (brown cells) and hematoxylin counterstained. (E) Duodenum of a vaccinated dog: on the right: the base of the villus: a goblet cell (GC) is included in the epithelium. Brush border (BB) is present at the apex of epithelial cells (EC); On the left: the subjacent interstitium is invaded by an immunocompetent cell infiltrate: plasma cells (PC) are numerous. LC, lymphocyte. (F) Duodenum of a control infected dog: on the right epithelial cells (EC) from the villus then basal lamina (BL). On the left the subjacent interstitium shows the presence of fibroblast (FC) and immunocompetent cells: plasma cell (Pl) and polynuclear cell (PN).

We observed CD3 labeling characterized by a strong staining of the cytoplasm of positive cells with reinforcement just beneath the cell membrane. CD3+ cells had small lymphocyte morphology. These cells were distributed predominantly in the upper half of the villus with decreasing density towards the base (Figure 1C and 1D)

In the lamina propria, the κ-, λ-, IgA-, and IgM-positive cells exhibited typical plasma cell morphology. The markers strongly and uniformly stained the cytoplasm of cells. We found considerably more λ-positive plasma cells than -positive cells (ratio λ/κ of 5), and IgA-containing plasma cells predominated with fewer IgM-positive cells (ratio IgA/IgM of 6.6). We observed the majority of IgA-containing cells located in the lower half of the villi (Figure 1A and 1B) and IgM-containing cells at the base of the villi.

Electron microscope (Figure 1E and 1F) and immunostaining results confirmed that immunized dogs mounted a strong inflammatory response compared to infected controls upon challenge. The interstitium was invaded by an immunocompetent cell infiltrate with numerous plasma cells and lymphocytes. We also identified many activated lymphocytes, characterized by their nucleus-cytoplasm ratio (<1), in Peyer's patches.

### Antibody responses against recombinant *Echinococcus* proteins

The quality of the immune response, and the pattern of the antibody subclass (IgG, IgA, IgE) were determined by ELISA. The IgA and IgE responses induced by the recombinant proteins were very low in all groups, and we observed no differences between the vaccinated group and controls.

## Discussion

To our knowledge, this is the first report of protection induced in dogs against the adult stage of *E. granulosus* after oral vaccination with a recombinant parasite protein. We have demonstrated the efficacy of the vaccine in two separate trials, each in a different country. Vaccination caused a decrease of more than 70% in the number of cestodes, and of a slower development rate of those recovered from vaccinated animals. Suppression of cestode development has also been observed recently [38] following the injection of a vaccine composed of recombinant EgM proteins. Out of consideration of the risk of infection to technical staff working with the patent infection in the dogs, we chose not to estimate the number of eggs in gravid segments, notwithstanding the opportunity offered by the strong expression of EgA31 in eggs and its potential usefulness as a marker [11]. We have demonstrated that a field vaccine based on this technology could be formulated into baits to target domestic and stray dogs in endemic countries.

Hydatid disease remains an important risk to human health and has a large economic impact. Several vaccine trials have thus been carried out in dogs, mainly using protoscolex membranes [8],[9] or excretory–secretory antigens from adult cestodes [18],[19]. These studies have demonstrated the ability of dogs to develop protective immunity against the development of adult *E. granulosus*. Recombinant proteins derived from a developmentally regulated gene family were recently used as a vaccine to reduce numbers of the adult cestode in dogs [10]. Among antigens evaluated as potential vaccines in sheep, conformational epitopes of recombinant Eg95 elicited the most protective immune response against the metacestode [19],[20].

Other proteins can induce a protective effect against helminthiases, including paramyosins [21]–[24] and tropomyosins [25],[26]. Analogs of those proteins are also present in *E. granulosus*. EgA31, a fibrillar protein presenting some properties of paramyosin, and tropomyosin (EgTrp) are both expressed in the metacestode and adult stages of *E. granulosus* [12],[27]. EgA31 is also strongly expressed in the immature strobilar stage of the parasite at the level of the tegument, parenchymal cells, and immature eggs [10], inducing an active immune response after injection, during the infection of dogs by *E.. granulosus* [10],[28]. Both EgA31 and EgTrp therefore seem to be promising antigens in the development of immunity against *E. granulosus* in dogs.

In the present study, these two recombinant proteins were expressed in a mutant of *S. typhimurium* that had been made avirulent in the dog, by precisely knocking out genes encoding enzymes in the prechorismate metabolic pathway [15]. This vaccine carrier strain has previously been used to express heterologous antigens and for effective oral immunization of dogs [15]. In 2004, Moreno al. demonstrated that the *S. typhimurium* carrier induces an immune response in dogs [29]. Studies in calves have shown that attenuated vaccines based on *S. typhimurium* or *S. dublin* calf isolates are capable of eliciting humoral and cellular immune responses, locally and at the systemic level, to *Salmonella* and heterologous antigens [30]–[33]. Using the plasmid pTECH, which has proven high stability as part of a *Salmonella* delivery system [13],[34], we expressed a short sequence of EgA31, including the most active epitopes [10], with the fusion partner TetC. Such *Salmonella* strains can confer partial protection against the pathogen from which the heterologous antigen was derived. In concordance with these previous findings, the dogs in the present study that received only the vector not expressing the antigen (group 2) showed a decrease in the number of worms compared to control.

Few studies have been published on the immuno-phenotypical characteristics of lymphoid cell populations in the normal canine gut. In accordance with previous studies on the topography of T cells [35],[36], we observed a greater number of CD3+ T cells at the top of the villi, whether the animals were infected or not. We chose not to compare the density of CD3+ T cells obtained in the present study with that previously reported, as these earlier studies focused on parasite-free animals [37]–[39].

Our immunostaining studies indicate a decrease of more than 50% in the number of T lymphocytes in the vaccinated dogs compared to all infected control groups, with the exception of dog number 4, which showed a different response to the vaccine.

Only one other study has reported a λ/κ ratio in the dog of around 10 in the lymph nodes [40]. In human lymphoid tissue, the λ/κ ratio is around 0.5. In our dog experiments the ratio is around 5 in the intestinal mucosa, and higher in the vaccinated dogs (5.6) compared to the infected control groups (4.9). Since the number of κ-positive cells is similar in all groups, the high λ/κ ratio observed in the vaccinated group could only be due to an increase in the number of λ-positive cells.

In the present study we detected no IgG or IgE antibodies. German et al. [35] observed a very small number (less than 15/mm^2^) of IgG-positive plasma cells in the lamina propria of the jejunum, and identified IgE positive cells as mast cells.^.^

In agreement with previous studies [35],[41], we observed a decrease in the number of plasma cells from the base to the top of the villi and identified the main secreted immunoglobulins as IgA. Our results indicate that the ratio of IgA to IgM is 6.9, whereas German et al. reported it as 9.7. The vaccinated dogs in our experiments showed a large increase in IgA-positive cells compared with dogs receiving only *Salmonella* (about 40%), though the density was similar to that in control dogs (about 5%). In light of our results we can verify a clear imbalance in the ratio of B to T lymphocytes in vaccinated dogs with a large decrease in the number of T cells and a small increase in the number of B cells, corresponding to IgA λ type–secreting plasma cells.

German et al. [35] hypothesized that T lymphocytes at the top of villi are of the Th1 lineage, whereas those at the base belong to the Th2 lineage. The decreased number of T lymphocytes at the top of the villi that we observed may explain the local humoral orientation of the immune response.

In contrast to the local humoral immune response, the ELISA results showed that the dogs developed no significant systemic humoral immune response against the EgA31-EgTrp vaccine. The low levels of IgG, IgA, and IgE detected in the serum showed no difference from levels in the negative control. However, the effect of the vaccine on the growth rate of the worms is clearly important at the intestinal level. This contradiction between the local and systemic immune responses after infection with *E. granulosus* has been studied and described by other groups [29],[42].

In conclusion, in all but one of our experimental dogs, the *Salmonella* vaccine EgA31-EgTrp showed efficacy against infection with and growth of *E. granulosus*. The vaccine has a local effect, leading to a decrease in the developing adult worm burden. Despite the fact that one dog apparently did not respond to the vaccine, we would suggest further development and testing in field trials. To decrease the number of worms remaining in vaccinated dogs, it could be interesting to integrate other recombinant proteins, such as those used by Zhang et al. [10], into our oral vaccine. Livestock vaccination reduces the infection pressure in the environment by decreasing the number of fertile larvae in intermediate hosts; the addition of an oral vaccine targeted at the adult stage of *E. granulosus* in dogs could help increase the overall efficacy of control programs in endemic countries.
