# Correction: An oral recombinant vaccine in dogs against *Echinococcus granulosus*, the causative agent of human hydatid disease: A pilot study

**DOI:** 10.1371/journal.pntd.0013238

**Published:** 2025-07-01

**Authors:** The PLOS Neglected Tropical Diseases Editors

After publication of this article [1], concerns were raised regarding the results presented in Fig 1, specifically:

- Fig 1E and Fig 1F appear similar despite representing different experimental conditions.
- In Fig 1D, two small regions just above the figure label appear similar.
- In Figs 1C and 1D, the areas surrounding the scale bars do not appear to match the surrounding areas, suggesting that these scale bars may have been pasted into the figure panels.

In 2017, PLOS discussed the Fig 1E-Fig 1F panel duplication concerns with the authors, who provided an updated figure (S1 File) in which panel E was replaced. Unfortunately, this case experienced a major delay and PLOS is no longer able to access the journal’s full 2017 correspondence records. The Fig 1C and Fig 1D concerns outlined above were not addressed in the original correspondence and were not clarified in recent discussions with the authors.

The authors indicated that the raw image data underlying Fig 1 are no longer available.

In addition, the limitations of the study design were not sufficiently addressed in the article’s Discussion section. This was a pilot study with relatively small treatment groups. The Materials and Methods section does not provide information about the vendors/sources from which animals were obtained, or about the breeds of dogs used in this study, baseline data, or whether the breeds and baseline data were well-matched across treatment conditions. It is possible that heterogeneity within and/or across groups may have affected the study outcomes. Furthermore, this study was not designed to evaluate the clinical efficacy of the vaccine. In light of these issues, the results about vaccine efficacy and protection afforded by the vaccine are not adequately supported and the study’s outcomes should be interpreted with caution. As is stated in the final paragraph of the Discussion section, one of the study’s conclusions is that further development and testing is needed.

The Editors note that the Materials and Methods section reports that dogs between 1–6 months of age were purchased and kept in facilities for 2 months before use. The age of animal #37 appears to be outside this age range based on the information reported in Table 1.

## Supporting information

S1 File Revised version of Figure 1. The updated figure presents the data at lower resolution than the original figure. Additionally, panels A-D in the revised version show different fields of view than the original published panels and do not include the areas of similarity within Fig 1D.
(TIF)

## References

[1] Petavy A-F, Hormaeche C, Lahmar S, Ouhelli H, Chabalgoity A, Marchal T, et al. An oral recombinant vaccine in dogs against *Echinococcus granulosus*, the causative agent of human hydatid disease: A pilot study. PLoS Negl Trop Dis. 2008;2(1):e125. doi: 10.1371/journal.pntd.0000125 18235847 PMC2217674

