# Supplementary figures and images for: Correction: An oral recombinant vaccine in dogs against Echinococcus granulosus, the causative agent of human hydatid disease: A pilot study
Source: PLoS Negl Trop Dis. 2025 Jul 1;19(7):e0013238. doi: 10.1371/journal.pntd.0013238 (PMC12212511; doi:10.1371/journal.pntd.0013238)

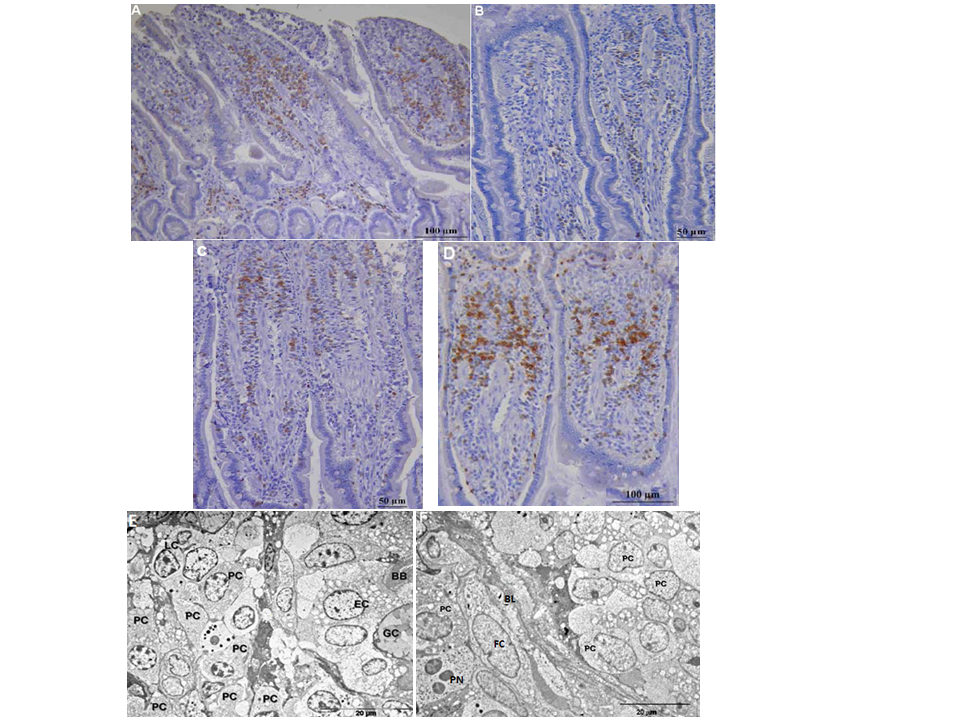

Supplement: S1 File — The updated figure presents the data at lower resolution than the original figure. Additionally, panels A-D in the revised version show different fields of view than the original published panels and do not include the areas of similarity within Fig 1D. (TIF) [file pntd.0013238.s001.tif]
